# Loss of STING impairs lactogenic differentiation

**DOI:** 10.1242/dev.202998

**Published:** 2024-10-14

**Authors:** Ramiah R. Vickers, Garhett L. Wyatt, Lilia Sanchez, Jordyn J. VanPortfliet, A. Phillip West, Weston W. Porter

**Affiliations:** ^1^ Department of Veterinary Physiology & Pharmacology, College of Veterinary Medicine, Texas A&M University, College Station, TX 77843, USA; ^2^ The Jackson Laboratory, Bar Harbor, ME 04609, USA

**Keywords:** STING, Mammary gland development, Lactation, Breast cancer, Inflammation, Mouse

## Abstract

Heightened energetic and nutrient demand during lactogenic differentiation of the mammary gland elicits upregulation of various stress responses to support cellular homeostasis. Here, we identify the stimulator of interferon genes (STING) as an immune supporter of the functional development of mouse mammary epithelial cells (MECs). An *in vitro* model of MEC differentiation revealed that STING is activated in a cGAS-independent manner to produce both type I interferons and proinflammatory cytokines in response to the accumulation of mitochondrial reactive oxygen species. Induction of STING activity was found to be dependent on the breast tumor suppressor gene single-minded 2 (SIM2). Using mouse models of lactation, we discovered that loss of STING activity results in early involution of #3 mammary glands, severely impairing lactational performance. Our data suggest that STING is required for successful functional differentiation of the mammary gland and bestows a differential lactogenic phenotype between #3 mammary glands and the traditionally explored inguinal 4|9 pair. These findings affirm unique development of mammary gland pairs that is essential to consider in future investigations into normal development and breast cancer initiation.

## Teaser

**Highlighted Article:** This work provides a new insight into how loss of the stimulator of interferon genes impairs lactogenic differentiation during normal mammary gland development.

## INTRODUCTION

Several lines of evidence have demonstrated that mitochondria have evolved to become activators of innate immunity (West and Shadel, 2017; West et al., 2011). Under conditions of stress or damage, mitochondria facilitate maintenance of cellular homeostasis by releasing their components into the intracellular space. Owing to its prokaryotic origin, mitochondrial components are recognized as foreign when exposed to innate immune receptors; thus, mitochondria are vital activators of host defense. The cGAS-STING (also known as STING1) pathway is a prominent innate immune pathway that has been widely explored in response to viral infection and mitochondrial damage. cGAS activation in response to cytosolic dsDNA results in STING-dependent production of type I interferons (IFNs) as well as proinflammatory cytokines. STING has also been implicated in several other signaling pathways that occur in a cGAS-independent manner, such as the DNA damage response (Dunphy et al., 2018), endoplasmic reticulum (ER) stress (Petrasek et al., 2013), autophagy (Gui et al., 2019) and metabolic reprogramming (Vila et al., 2022); ultimately, supporting its role in maintaining cellular homeostasis. Currently, knowledge of how STING facilitates homeostasis during normal tissue development remains limited. The mouse mammary gland provides a unique model to address this gap in knowledge, as the gland undergoes extensive postnatal development.

During pregnancy, as mammary epithelial cells (MECs) differentiate into milk-secreting structures, they undergo a metabolic transition and alter their gene expression profiles to favor impending increases in energy production (Elswood et al., 2021; Sanchez et al., 2023). These changes result in lactating MECs possessing longer, more fused mitochondria that have more efficient electron transport chain (ETC) super complex formation to support cell survival during this time of metabolic demand (Cogliati et al., 2013; Elswood et al., 2021; Gomes et al., 2011; Mishra et al., 2014; Sanchez et al., 2023). This increase in energetic demand ultimately challenges mitochondrial homeostasis, as the newly formed mitochondrial network is more susceptible to increased fatigue and generation of harmful reactive oxygen species (ROS). Following cellular insult, such as accumulation of mitochondrial ROS (mtROS), inflammation is enhanced to promote tissue regeneration, therefore, resulting in restoration of cellular homeostasis. Cancer is often described as a wound that fails to heal (Coussens and Werb, 2002; Dvorak, 1986); thus, we predicted that failure of an inflammatory modulator such as STING could promote tumor development under the severe metabolic distress of lactation. Here, we propose that STING activity is required to sustain cellular homeostasis during MEC differentiation to promote successful lactation. Furthermore, we predict that a loss of STING activity within mammary tissue predisposes the gland to tumor development. We anticipate that investigating STING during lactogenic differentiation will provide more comprehensive knowledge of how it contributes to normal mammary gland development and the onset of disease.

To address how STING contributes to lactogenic differentiation, we employed an *in vitro* model of MEC differentiation and assessed activation of STING. We paired this with a loss-of-function of STING mouse model, the STING Goldenticket (*Sting^gt/gt^*), and observed that a loss of STING activity results in a failure of lactogenic differentiation in #3 mammary glands, ultimately impairing lactational performance and promoting breast cancer initiation. We propose that a STING-dependent inflammatory balance supports MEC functionality under conditions of severe metabolic stress during lactation. Ultimately, this work provides important insight into how STING activation within differentiating MECs influences functional lactation and also has innovative insight into normal development of the mammary gland as well as breast cancer initiation.

## RESULTS

### Lactation induces STING expression in a cGAS-independent manner

**Fig. 1.**
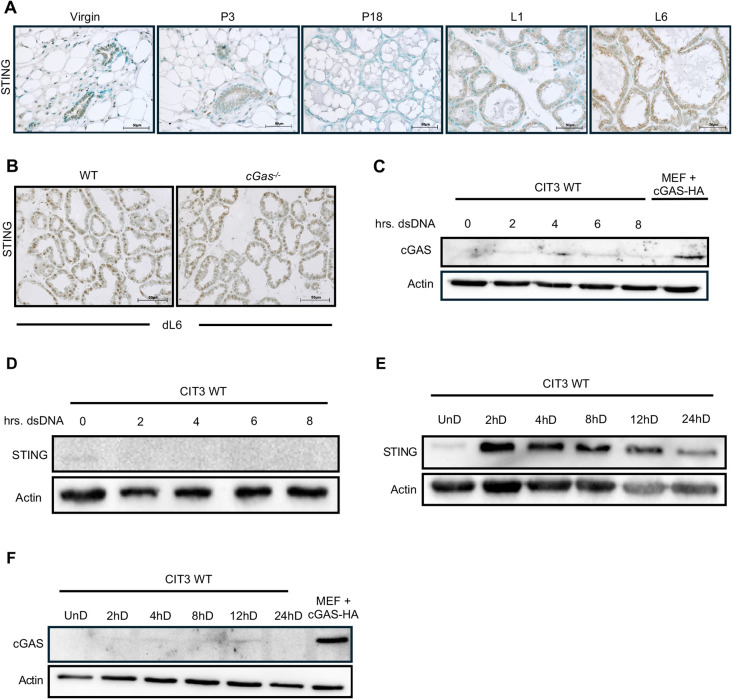
**cGAS-independent STING expression during mammary epithelial cell differentiation.** (A) Immunostaining for STING in #4 mammary glands of WT mice across normal development; P, pregnancy day; L, lactation day. (B) Immunostaining for STING in *cGas*^−/−^ mammary gland tissue at lactation day (dL) 6. (C,D) Immunoblot analysis of cGAS (C) and STING (D) in undifferentiated CIT3 WT cells transfected with dsDNA. (E,F) Immunoblot analysis of STING (E) and cGAS (F) in CIT3 WT cells across MEC differentiation. Mouse embryonic fibroblasts (MEFs) transfected with cGAS-HA used as positive control for cGAS expression. hD, hours of differentiation. Scale bars: 50 µm.

cGAS-STING pathway activation has been widely explored in response to mitochondrial damage. This knowledge led us to hypothesize that metabolic stress during lactation could serve as an activator of cGAS-STING signaling. To determine whether mammary epithelial cells trigger cGAS-STING activation, we performed immunohistochemical analysis of STING protein expression across mammary gland development. We found that STING expression is low in the virgin gland and during early pregnancy, and by pregnancy day 18, STING expression was diminished (Fig. 1A). Of interest, with the onset of lactation, STING expression dramatically increased and was exacerbated as lactation continued at day 6. It has been established that mtROS can trigger STING activity in a cGAS-independent manner (Mills et al., 2017). To explore this mechanism during lactation, we examined STING expression in *cGas*^−/−^ mammary glands. Interestingly, we observed comparable STING expression at lactation day 6 in wild-type (WT) and *cGas*^−/−^ mammary glands (Fig. 1B), suggesting that STING functions in a cGAS-independent manner within the lactating mammary gland. Based on these data, we moved to an *in vitro* model to further explore STING activity during MEC differentiation.

**Fig. 2.**
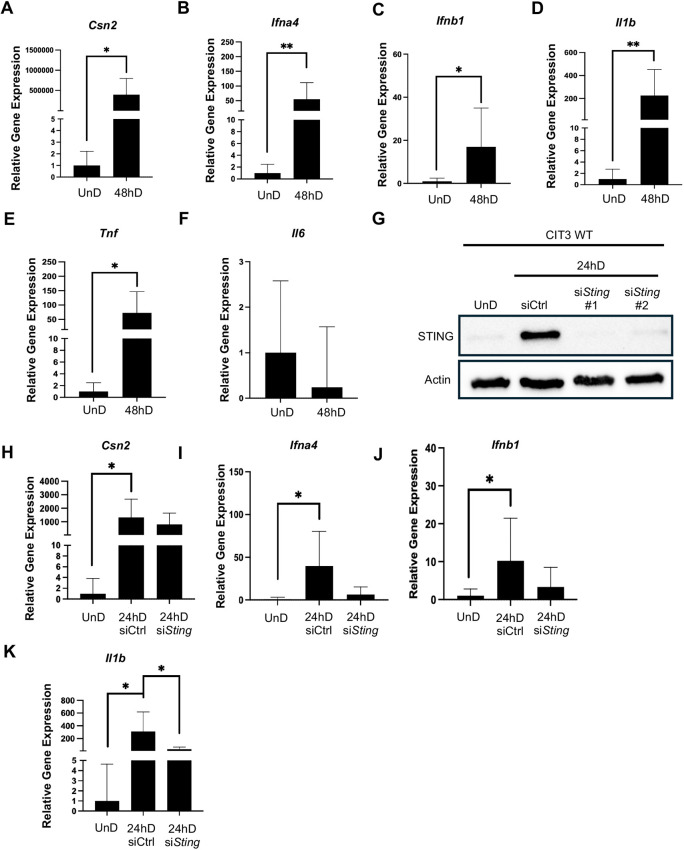
**STING inhibition reduces type I IFN gene expression during MEC differentiation.** (A-F) qPCR analysis of *Csn2* (A), *Ifna4* (B), *Ifnb1* (C), *Il1b* (D), *Tnf* (E) and *Il6* (F) in CIT3 WT, undifferentiated (UnD) versus 48 h of differentiation (hD). (G) Immunoblot analysis of CIT3 WT cells transfected with si*Sting* at 24 hD. (H-K) qPCR analysis of *Csn2* (H), *Ifna4* (I), *Ifnb1* (J) and *Il1b* (K) in CIT3 WT cells following si*Sting* transfection at 24 hD. *P≤0.05, **P≤0.01 (paired, two-sided Student's *t*-test). Data are mean±s.d.

We first wanted to confirm a lack of cGAS activity within MECs *in vitro*. To measure cGAS function, we transfected CIT3 mammary epithelial cells in their undifferentiated (UnD) state with a short 90 bp fragment of dsDNA over the course of several hours. This DNA fragment would serve as a ligand for cGAS, a cytosolic DNA sensor, resulting in its activation. Immunoblot analysis revealed a lack of cGAS expression in response to dsDNA transfection (Fig. 1C); however, we observed low basal STING expression (Fig. 1D). As previously mentioned, MECs differentiate to produce milk-secreting structures that are vital for functional lactation. Upregulation of prolactin and progesterone during pregnancy trigger rapid proliferation and differentiation of MECs. *In vitro*, the addition of prolactin and hydrocortisone, along with the removal of epidermal growth factor (mEGF), marks the initiation of MEC differentiation. The absence of hormones is denoted as the cells in their UnD or virgin-like state. To evaluate cGAS-STING signaling in response to hormonal cues, we differentiated CIT3 WT cells. We observed low STING expression in UnD cells which increased in response to hormones after 2 h of differentiation (hD) (Fig. 1E). This result indicates that STING is rapidly activated in response to hormonal cues. STING expression remained high though 12 hD and started to subside after 24 hD. We did not observe cGAS expression in response to hormones (Fig. 1F), further confirming cGAS-independent STING expression during MEC differentiation. Next, we wanted to assess induction of downstream type I IFNs and several proinflammatory cytokines. We first validated that the cells were differentiating properly by assessing gene expression of *Csn2*, responsible for the production of the milk protein β-casein. We observed a significant increase in *Csn2* expression at 48 hD (Fig. 2A). Moreover, we observed significant increases in the type I IFNs, *Ifna4* (Fig. 2B) and *Ifnb1* (Fig. 2C). We also observed significant increases in gene expression of the proinflammatory cytokines *Il1b* (Fig. 2D) and tumor necrosis factor (*Tnf*) (Fig. 2E); however, we did not observe any significant changes in expression of *Il6* (Fig. 2F). These results confirm that both type I IFN and pro-inflammatory signaling are present during MEC differentiation.

To confirm that STING activity is responsible for type I IFN gene expression in response to differentiation cues, we implemented use of a small interfering RNA construct to inhibit *Sting* (si*Sting*). Western blot analysis confirmed a loss of STING protein expression at 24 hD following transfection of si*Sting* constructs (Fig. 2G). We noted a modest decrease in *Csn2* gene expression following STING inhibition (Fig. 2H). Furthermore, we observed decreases in gene expression of *Ifna4* (Fig. 2I) and *Ifnb1* (Fig. 2J). Moreover, we found that STING inhibition significantly decreased gene expression of the proinflammatory cytokine *Il1b* (Fig. 2K). Prolonged exposure to type I IFNs as well as proinflammatory cytokines is detrimental to the host and is also known to promote cancer development (Crow, 2011; Davidson et al., 2015), which is a plausible reason for the decrease in STING expression as MECs progress throughout differentiation. This reinforces our hypothesis that STING facilitates the maintenance of cellular homeostasis throughout MEC differentiation via induction of an inflammatory gene profile. Ultimately, STING facilitates induction of type I IFN and proinflammatory signaling in an immune regulator factor 3 (IRF3)- and nuclear factor-κB (NF-κB)-dependent manner, respectively; thus, this suggests multiple mechanisms of downstream STING signaling during MEC differentiation.

### Loss of SIM2 decreases STING expression

**Fig. 3.**
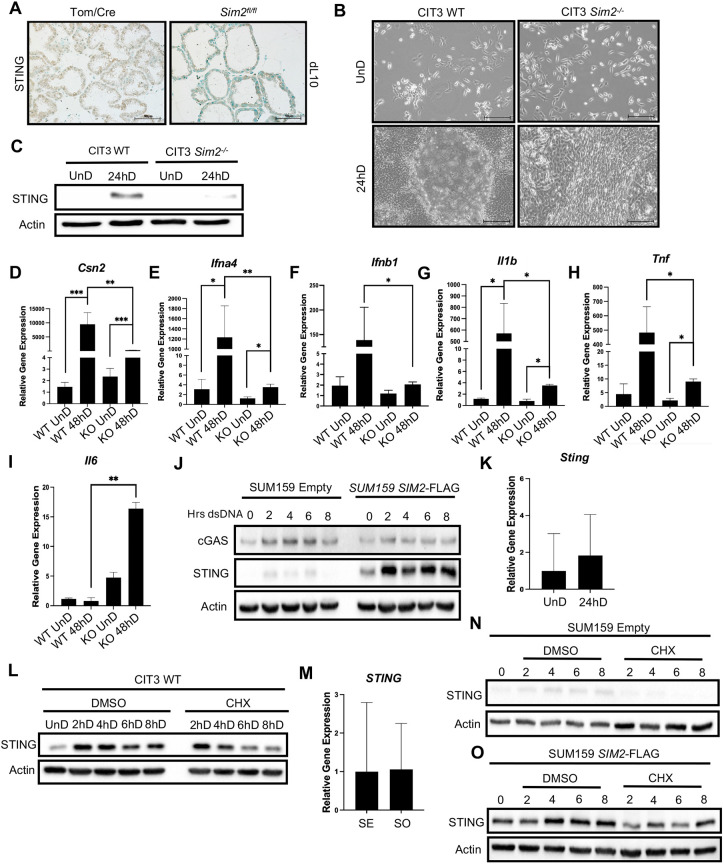
**Loss of *Sim2* decreases STING expression.** (A) Immunostaining for STING in control and *Sim2^fl/fl^* #4 mammary glands at lactation day (dL) 10. (B) Microscopy images of undifferentiated (UnD) versus 24 h of differentiation (hD) CIT3 WT and *Sim2*^−/−^ MECs. (C) Immunoblot analysis of STING in CIT3 WT and *Sim2*^−/−^ MECs. (D-I) qPCR analysis of *Csn2* (D), *Ifna4* (E), *Ifnb1* (F), *Il1b* (G), *Tnf* (H) and *Il6* (I) in CIT3 WT and *Sim2*^−/−^ cells. (J) Immunoblot analysis of cGAS-STING in SUM159 cells following dsDNA transfection. (K) qPCR analysis of Sting in CIT3 WT cells. (L) Immunoblot analysis of CIT3 WT cells following CHX treatment across early differentiation. (M) qPCR analysis of STING in SUM159 cells. (N,O) STING protein expression following CHX treatment in SUM159 Empty (N) and *SIM2*-overexpressing (O) cells. *P≤0.05, **P≤0.01, ***P≤0.001 (paired, two-sided Student's *t*-test). Data are mean±s.d. Scale bars: 50 µm.

It has previously been shown that single-minded 2 (SIM2) functions as a tumor suppressor within the mammary gland, as we observed a loss of *Sim2* expression with progression to invasive ductal carcinoma (Pearson et al., 2019; Scribner et al., 2013). Furthermore, we have demonstrated that a loss of *Sim2* resulted in aberrant ductal development as well as evidence of an epithelial-to-mesenchymal transition (EMT), characterized by a loss of polarity, increased proliferation and invasion of the basement membrane (Laffin et al., 2008). More recently, we have established that SIM2 serves as a sensor of cellular stress during MEC differentiation. Ultimately, SIM2 facilitates the functional success of MECs during lactogenic differentiation by enhancing mitophagy, the clearance of damaged mitochondria (Elswood et al., 2021; Sanchez et al., 2023). Given this knowledge, we also hypothesized that SIM2 could enhance innate immune activation as a mechanism to support MEC survival during lactation. To investigate the involvement of SIM2 in STING-mediated immunity during MEC differentiation, we first evaluated STING expression in a model of conditional *Sim2* loss within the mammary gland via a floxed allele (*Sim2^fl/fl^*). We found that a loss of *Sim2* greatly diminished STING expression during peak lactation (day 10) (Fig. 3A), suggesting that SIM2 is a potential regulator of STING during lactation. These data also confirm that elevated STING expression continues throughout lactation. To further investigate the relationship between SIM2 and STING, we generated *Sim2*^−/−^ CIT3 cells using CRISPR-Cas9. Imaging did not reveal notable differences between WT and *Sim2*^−/−^ cells in their undifferentiated state; however, in response to differentiation hormones, *Sim2*^−/−^ cells displayed a vastly different morphology compared with WT cells (Fig. 3B). At 24 hD, WT cells form large, dome-like structures that are multi-planar. In contrast, *Sim2*^−/−^ cells remain in a single plane and exhibit a more basal-like phenotype that is characteristic of an EMT. To validate these cells, we performed subcellular fractionation and confirmed a loss of *Sim2* within the mitochondrial fraction (Fig. S1). Further analysis revealed that STING expression was greatly diminished at 24 hD with a loss of *Sim2* (Fig. 3C), further alluding to a dynamic relationship between SIM2 and STING. Next, we confirmed significant decreases in gene expression of *Csn2* (Fig. 3D), *Ifna4* and *Ifnb1* (Fig. 3E,F) as well as *Il1b* (Fig. 3G) and *Tnf* (Fig. 3H) with a loss of *Sim2*. Interestingly, there was a significant increase in *Il6* at 48 hD with loss of *Sim2* compared with WT cells (Fig. 3I). These data indicate that loss of *Sim2* negatively impacts STING activity during MEC differentiation and may, as a result, also confer differential cytokine activation.

Accumulating evidence has highlighted the emergence of STING signaling as an attractive focal point in cancer therapeutics (Chen et al., 2020; Corrales et al., 2015; Kulasinghe et al., 2021; Shen et al., 2022; Zhang et al., 2022). Given that STING signaling is suppressed in most cell types (Konno et al., 2018; Ying-Rui et al., 2023), immunological enhancement of STING activity has been demonstrated to be a promising route for cancer immunotherapy. To further investigate the relationship between SIM2 and STING, we employed use of a model of triple negative breast cancer (TNBC). We used SUM159 cells as a model system, as they do not express basal levels of SIM2, allowing us to overexpress *SIM2* to investigate its functional roles in cancer (Kwak et al., 2007; Sanchez et al., 2023; Scribner et al., 2013; Wall et al., 2023; Wyatt et al., 2019). Additionally, postpartum breast cancer (PPBC) tumors are highly aggressive and approximately three times more likely to metastasize (Goddard et al., 2019), thus, we opted for a TNBC model as these tumors also demonstrate increased metastatic potential and a lack of beneficial therapeutic intervention (Li et al., 2022). To confirm cGAS-STING activation in the SUM159 model, we transfected dsDNA into SUM159 control and *SIM2*-overexpressing cells. In control cells with low levels of SIM2, we observed induction of cGAS after 2 h of dsDNA transfection, which persisted through 6 h of transfection (Fig. 3J). After 8 h, cGAS expression began to diminish. Concurrently, we observed very slight induction of STING following dsDNA transfection between 2 and 6 h. Interestingly, when *SIM2* is overexpressed, cGAS is not induced following transfection of dsDNA; however, we observed robust induction of STING following dsDNA transfection that persisted through 8 h. These data suggest that SIM2 is involved in the regulation of STING in a cGAS-independent manner within mammary tissue. A recent study has uncovered that TNBC patients that are responsive to chemotherapeutic treatments exhibited increased STING protein expression (Kulasinghe et al., 2021); therefore, further explorations into SIM2 regulation of STING may be beneficial to predict responsiveness to immunotherapeutic treatment for women afflicted with breast cancer.

As a member of the innate immune system, STING is a part of the host's first line of defense. Innate immune reactions are non-specific and therefore must occur rapidly, within minutes or hours, following the insulting agent. Although STING activity has been extensively explored and defined, STING regulation has yet to be fully elucidated (Kuchitsu et al., 2023). We found that STING gene expression was not significantly altered in CIT3 WT cells in response to hormonal cues at 24 hD (Fig. 3K); thus, we anticipated that STING is largely regulated independently of transcriptional control within the immediate stage of its activation. To evaluate whether STING is expressed independently of novel protein synthesis, we treated CIT3 WT cells with cycloheximide (CHX) over the course of several hours to inhibit translation. Immunoblot analysis revealed that STING remained highly expressed following 2 h CHX treatment, although protein expression was diminished as CHX treatment persisted (Fig. 3L). This evidence demonstrated that STING protein expression is largely independent of transcriptional regulation during MEC differentiation. Conversely, in most cancer cell types, STING is transcriptionally suppressed through either loss-of-function mutations or epigenetic silencing (Konno et al., 2018; Ying-Rui et al., 2023; Zhang et al., 2022). In our SUM159 model, we demonstrated that overexpression of *SIM2* enhances STING expression at the protein level, thus, we wanted to further explore how SIM2 regulates STING. qPCR analysis revealed no changes in STING RNA expression between control and *SIM2*-overexpressing cells (Fig. 3M). We next subjected these cells to CHX treatment. Under basal conditions, control cells with low levels of SIM2 expressed very low levels of STING, which were almost completely diminished following CHX treatment (Fig. 3N). In our *SIM2*-overexpressing model, STING expression was basally high and remained elevated through 8 h of CHX treatment (Fig. 3O). These results strongly suggest that SIM2 serves to stabilize STING protein expression in a model of TNBC.

### Mitochondrial ROS as an activator of STING during MEC differentiation

**Fig. 4.**
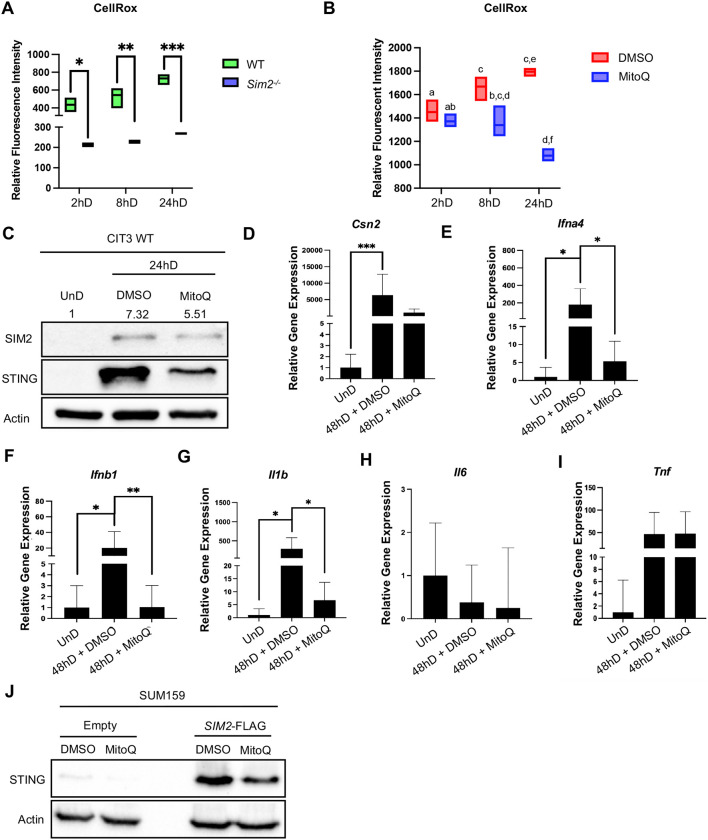
**Mitoquinol treatment decreases STING-dependent gene expression.** (A) Relative fluorescence intensity of cellular reactive oxygen species (ROS) in CIT3 WT versus *Sim2*^−/−^ cells at 2, 8 and 24 h of differentiation (hD). (B) Relative fluorescence intensity of cellular ROS in CIT3 WT cells following MitoQ treatment. In A,B, boxes represent mean±s.d. (C) Immunoblot analysis of SIM2 and STING protein expression in CIT3 WT cells at 24hD, with and without MitoQ treatment. Numbers above the blot represent densitometry analysis for SIM2. (D-I) qPCR analysis of *Csn2* (D), *Ifna4* (E), *Ifnb1* (F), *Il1b* (G), *Il6* (H) and *Tnf* (I) in CIT3 WT cells at 48 hD, with and without MitoQ treatment. (J) Immunoblot analysis of SUM159 Empty and *SIM2*-FLAG cells following 24 h MitoQ treatment. *P≤0.05, **P≤0.01, ***P≤0.001 (paired, two-sided Student's *t*-test). Data are mean±s.d.

Under conditions of cellular stress, mtROS has been established to act as a danger signal that promotes host defense and inflammation (Chen et al., 2021; Jin et al., 2017; Martinon et al., 2009; Mills et al., 2017; Mittal et al., 2014; Sandhir et al., 2017; Smith, 2020; Swanson et al., 2019). As a result, maintenance of mitochondrial homeostasis is essential to mediate innate immunity. Owing to heightened energetic demand during lactation, we hypothesized that STING activity during MEC differentiation is a result of ROS accumulation from fatigued mitochondria. We have previously published that SIM2 promotes oxidative phosphorylation (OXPHOS) via direct interaction with components of the mitochondrial respiratory chain, ultimately facilitating super complex formation (Wall et al., 2023). These data suggest that SIM2 indirectly promotes the generation of mtROS, which could be considered as a potential mechanism of how SIM2 enhances STING activity. To better understand the mechanism of how SIM2 regulates STING, we measured the amount of cellular ROS in WT and *Sim2*^−/−^ MECs. We found that loss of *Sim2* conferred a significant decrease in cellular ROS throughout MEC differentiation (Fig. 4A). Next, we explored whether a decrease in ROS would decrease STING expression. To first confirm a decrease in ROS levels, we treated CIT3 WT cells with a mtROS-specific scavenger, mitoquinol (MitoQ), over the course of MEC differentiation. We confirmed that ROS increases significantly as MECs differentiate and that this accumulation of ROS is mitigated with MitoQ treatment (Fig. 4B). Immunoblot analysis revealed that both SIM2 and STING expression were decreased at 24 hD with addition of MitoQ treatment, suggesting that induction of SIM2 and STING is elevated in response to mtROS production (Fig. 4C). This evidence complements our findings that SIM2 also functions as a cellular sensor and responds to stress (Sanchez et al., 2023).

It has been previously reported that ROS facilitates cellular differentiation of mammary epithelial cells (Baratta et al., 2018; Elswood et al., 2021); we therefore investigated whether a decrease in ROS would impact STING-dependent gene expression. qPCR analysis revealed that MitoQ treatment decreased gene expression of *Csn2* (Fig. 4D), indicative that a decrease in mtROS production negatively impacted the functional performance of MECs. Additionally, we observed significant decreases in both *Ifna4* (Fig. 4E) and *Ifnb1* (Fig. 4F) with a reduction in mtROS, suggesting that the type I IFN response during MEC differentiation is, in part, reliant upon mtROS signaling. We also noted a significant decrease in *Il1b* gene expression (Fig. 4G). As previously seen, we did not observe significant alterations in gene expression of *Il6* (Fig. 4H). Interestingly, we did not observe changes in gene expression of *Tnf* following MitoQ treatment (Fig. 4I), suggesting that *Tnf* gene expression may be resultant from other mechanisms. Ultimately, dysregulation between mitochondrial quality control mechanisms and proinflammatory signaling can fuel a cycle of ROS accumulation that will augment inflammation to support maintenance of cellular homeostasis; however, aberrant activation of inflammatory signaling has been demonstrated to promote the development of several inflammatory diseases such as cancer (Mittal et al., 2014; Schulz et al., 2000). Of note, failure of inflammatory signaling also contributes to disease development due to a disruption of homeostasis; therefore, we propose that immune modulators such as STING are essential to maintaining a delicate inflammatory balance during lactogenic differentiation of the mammary gland.

Several lines of evidence have demonstrated that ROS dynamically influences the tumor microenvironment, ultimately promoting the metastasis and survival of tumor cells (Aggarwal et al., 2019; Mittal et al., 2014). Moreover, accumulating oxidative damage fosters an inflammatory environment, leading to innate immune intervention. Aberrant or failure of inflammatory pathways ultimately promotes tumor development and progression; thus, innate immune pathways are appealing targets in current immunotherapeutic treatments. For example, STING enhancement has proven to be beneficial for the treatment of some cancers, although STING agonists do not have inhibitory effects on all tumor types. In some cancers, STING expression has actually been shown to foster a niche for carcinogenesis (Zhang et al., 2022), although the mechanism of action is still largely unknown. We hypothesized that aberrant STING expression when *SIM2* is overexpressed in a model of TNBC could be pro-tumorigenic; thus, diminishing STING expression could be of potential interest in therapeutic treatment. To investigate whether ROS accumulation as a result of *SIM2* overexpression resulted in increased STING levels, we subjected SUM159 cells to MitoQ treatment for 24 h. Decreased mtROS production substantially diminished STING expression in control cells and moderately decreased STING protein levels when *SIM2* was overexpressed (Fig. 4J). These data suggest that mitigation of mtROS accumulation is a sufficient mechanism to diminish STING expression when *SIM2* is overexpressed; however, additional mechanisms must also be explored in relation to SIM2 regulation of STING activation.

### Loss of STING activity impairs lactational performance

To continue our efforts in investigating the contribution of STING to mammary gland development, we used a loss-of-function STING mouse model, the STING Goldenticket (*Sting^gt/gt^*). The *Sting^gt/gt^* harbors a chemically induced missense mutation in exon 6 resulting in an isoleucine-to-asparagine change in amino acid 199 in the C-terminal region (Sauer et al., 2011). Multiple doses of N-ethyl-N-nitrosourea (ENU) were used to induce mutations before a forward genetic screen to identify mice exhibiting the desired mutation, which was determined to result in ablation of STING-mediated immunity (Sauer et al., 2011). Primary analysis of #4 mammary gland architecture via Hematoxylin and Eosin (H&E) staining of 10-week virgins suggested a minor reduction in alveolar quantity in *Sting^gt/gt^* mice; however, no notable differences in alveolar structure were seen (Fig. S2A). Following examination of pregnancy day 16 glands, we determined that there were fewer notable differences in alveolar quantity, although at this time we began to discern variations in alveolar expansion in *Sting^gt/gt^* mice (Fig. S2A). Dams collected at lactation day 10 revealed a much more substantial reduction in lactogenic expansion of the mammary gland, with a loss of STING activity when compared with WT glands (Fig. S2A). Despite the presence of smaller alveoli in *Sting^gt/gt^* mice, we measured an increased number of nuclei present in these glands (Fig. S2B). We anticipated that an increased number of cells might influence milk production; however, the decreased size of the lobuloalveoli led us to investigate expression of CSN2 to evaluate the amount of milk production in these mice. Immunohistochemical analysis showed significantly decreased expression of CSN2 in *Sting^gt/gt^* dams (Fig. S2C,D), suggesting that these mice have an impaired ability to produce milk. This phenomenon ultimately provided motive to explore lactational performance within *Sting^gt/gt^* mice.

**Fig. 5.**
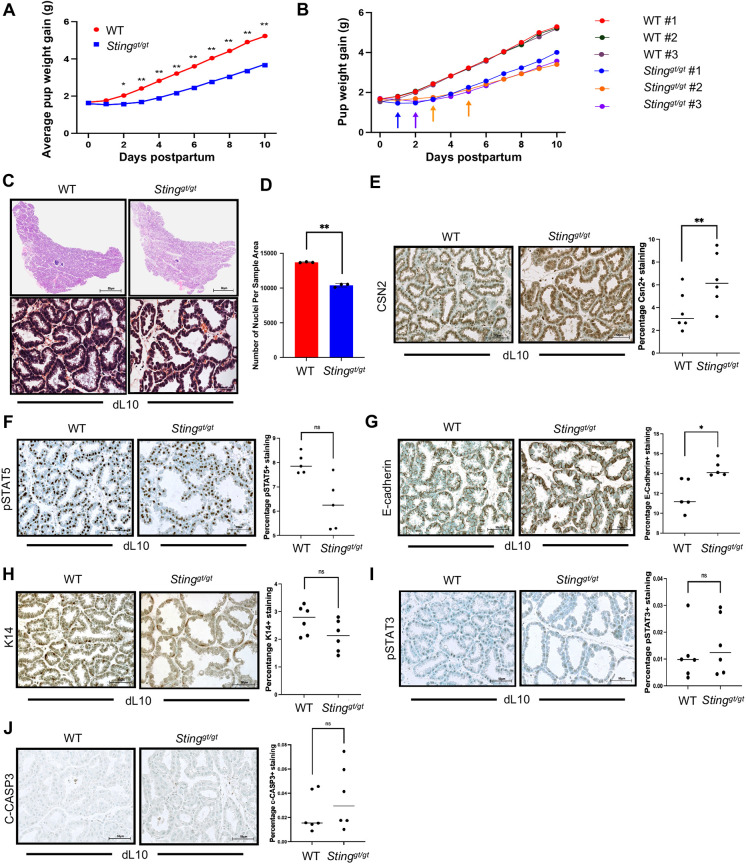
**Loss of *Sting* negatively impacts lactational performance.** (A) Average weight of cross-fostered pups nursed by WT and *Sting^gt/gt^* mice (*n*=3 WT, *n*=3 *Sting^gt/gt^*). (B) Weight of cross-fostered pups nursed by WT and *Sting^gt/gt^* mice. Arrows indicate death of pup. (C) H&E-stained #4 mammary tissue sections from WT and *Sting^gt/gt^* mice at lactation day (dL) 10. (D) Quantification of cellularity (number of nuclei per sample area) for WT and *Sting^gt/gt^* mice. (E-J) Immunostaining and quantification of percentage of staining at dL10 for CSN2 (E) pSTAT5 (F) E-cadherin (G) K14 (H) pSTAT3 (I) and c-CASP3 (J). *P≤0.05, **P≤0.01 (paired, two-sided Student's *t*-test). Data are mean±s.d. Scale bars 50 µm.

Our lab has recently uncovered that SIM2 contributes to MEC differentiation by facilitating the rapid turnover of mitochondria during lactation, ultimately influencing lactational performance. Specifically, we found that loss of *Sim2* negatively impacted lactation, whereas overexpression of *Sim2* enhanced lactation (Sanchez et al., 2023). As previously demonstrated, a loss of *Sim2* confers a loss of *Sting*. Given this evidence, taken together with decreased CSN2 expression in *Sting^gt/gt^* mice, we wanted to explore how loss of STING activity impacts MEC function. To address how STING impacts lactational performance, we measured the weight gain of pups nursed by WT and *Sting^gt/gt^* dams. To eliminate any potential bias derived from the genotype of the pups, we used ten weight and age-matched pups from donor ICR WT mice per dam. We found that pups nursed by *Sting^gt/gt^* dams gained significantly less weight than those nursed by WT mice by day 2 of lactation, and this deficit persisted throughout mid-lactation (Fig. 5A). Of interest, *Sting^gt/gt^* dams lost pups throughout the duration of the experiment, demonstrating that they were unable to sustain functional lactation (Fig. 5B). Therefore, the fatality and decreased weight of pups nursed by *Sting^gt/gt^* mice suggests that loss of STING activity negatively impacts lactogenic differentiation.

Following cross-fostering, we reanalyzed mammary gland architecture using H&E staining. We observed similar characteristics of smaller lobuloalveoli and impaired cell adhesion as observed before cross-fostering (Fig. 5C). Unlike before, we found that #4 glands from *Sting^gt/gt^* dams exhibited significantly fewer cells per sample area (Fig. 5D). Reassessment of CSN2 protein uncovered that challenging lactational capacity decreased the amount of milk in the luminal compartment in WT dams (Fig. 5E). Furthermore, CSN2 expression is increased in *Sting^gt/gt^* dams, which was inconsistent with previous data. This suggests that the decrease in weight gain observed in pups nursed by *Sting^gt/gt^* dams is likely independent of the amount of overall CSN2 production within the gland. Signal transducer and activator of transcription 5 (STAT5) is required to promote the development of lobuloalveoli and is also a vital transcriptional regulator of milk proteins, including CSN2 (Liu et al., 1997; Wakao et al., 1994). In response to prolactin and several interleukins (IL2, IL3, IL5, IL7, IL9 and IL15), STAT5 is phosphorylated, leading to its activation and expression of milk protein genes (Barash, 2006). To confirm that CSN2 expression was not impacted by transcriptional regulation, we assessed expression of pSTAT5 and did not find any notable differences between WT and *Sting^gt/gt^* dams (Fig. 5F). These results suggest that loss of STING activity does not negatively affect the activation of STAT5 or the development of lobuloalveoli. This signifies that the decrease in weight observed in pups nursed by *Sting^gt/gt^* dams cannot be attributed to impaired transcriptional regulation of milk proteins.

Based on this evidence, we began to speculate about the primary differentiation status of mammary epithelial cells with a loss of STING activity. Hormonal signals during mammary gland development initiate evolution to a complex architecture heavily dependent upon strong cell adhesion. It has been established that E-cadherin, the major cadherin expressed in luminal epithelial cells, is required for terminal MEC differentiation and structural maintenance of alveoli within the lactating mammary gland (Shamir and Ewald, 2015). Specifically, loss of E-cadherin triggers lobuloalveolar collapse in addition to massive apoptosis (Boussadia et al., 2002). Moreover, loss of E-cadherin is a classical indicator of an EMT; therefore, as a key adhesion molecule, E-cadherin is regarded as a suppressor of tumor invasion and metastasis in various epithelial cancers (Beavon, 2000). H&E analysis of *Sting^gt/gt^* dams revealed a potential disruption in cell adhesion between luminal epithelial cells (Fig. 5C); thus, we evaluated expression of E-cadherin to assess structural integrity. We found that *Sting^gt/gt^* dams exhibited higher expression of E-cadherin compared with WT controls (Fig. 5G). In dispute of the concept that E-cadherin suppresses metastasis, it has been revealed that a cohort of breast tumors, as well as their metastases, are actually positive for E-cadherin (Kowalski et al., 2003). Moreover, studies have shown that E-cadherin expression can be ‘normal’ or augmented in models of inflammatory breast cancer, making our results in *Sting^gt/gt^* mice of interest in regard to breast cancer initiation (Kleer et al., 2001). This paradox between findings could be resultant of transient loss of E-cadherin during metastatic migration (Polyak and Weinberg, 2009). Analysis of cytokeratin 14 (K14; Krt14), prominently expressed in myoepithelial cells, revealed no gross differences (Fig. 5H). These data ultimately led us to consider other avenues for pup fatality and decreased weight gain as a result of a loss of STING activity. To rule out the possibility of premature involution, we evaluated expression of the involution marker pSTAT3 and did not observe any notable differences (Fig. 5I). Moreover, we did not observe an increase in the cell death marker, cleaved caspase 3 (c-CASP3) in *Sting^gt/gt^* dams (Fig. 5J). These findings indicate that the decreased weight gain and loss of pups nursed by *Sting^gt/gt^* dams cannot be attributed to premature involution or an increase in cell death, both of which would greatly diminish MEC quantity. Altogether, this evidence suggests that some other dynamic is contributing to lactation impairment in *Sting^gt/gt^* dams.

### Loss of STING results in early involution of #3 mammary glands

Previous studies have shown that the five pairs of mammary glands within the mouse are each produced by unique signaling networks (Veltmaat et al., 2013). Furthermore, despite their morphological bilateral symmetry, left-right somites have significant differences in gene expression (Golding, 2004a,b). Although the murine inguinal pair 4|9 is widely used for molecular and histological analysis, it has been determined that pairs 2|7 and 3|8 of the superior position correspond more closely with that of the human breast (Veltmaat et al., 2013) (Fig. S3A). Additionally, inflammatory signaling and tumor growth have been established to be more prominent within superior regions (Auerbach and Auerbach, 1982), rendering them more interesting in terms of translational application. Histological analysis of the inguinal #4 gland did not fully explain the rationale behind the loss and decrease in weight gain of pups nursed by *Sting^gt/gt^* dams; thus, we determined that investigating a more superior gland would bestow clarity in addition to comparativeness with the human gland. Interestingly, we found striking disparities in the #3 mammary glands following cross-fostering experiments that likely explain the decreased weight and death of pups nursed by *Sting^gt/gt^* dams that was previously unaccounted for by assessing inguinal #4 glands (Fig. S3B,C).

**Fig. 6.**
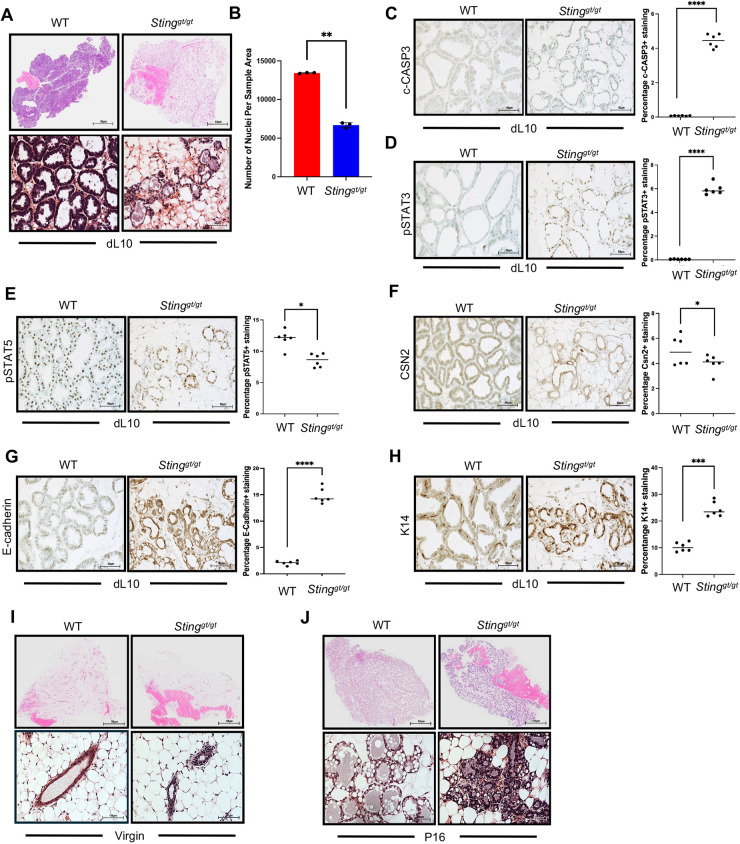
**Loss of *Sting* impairs functional differentiation in #3 mammary glands.** (A) H&E-stained #3 mammary tissue sections from WT and *Sting^gt/gt^* mice at lactation day (dL) 10. (B) Quantification of cellularity (number of nuclei per sample area) for WT and *Sting^gt/gt^* mice. (C-H) Immunostaining at dL10 for c-CASP3 (C), pSTAT3 (D), pSTAT5 (E), CSN2 (F), E-cadherin (G) and K14 (H). (I,J) H&E-stained #3 mammary tissue sections from WT and *Sting^gt/gt^* mice at 10-week virgin (I) and pregnancy day (P)16 (J). *P≤0.05, **P≤0.01, ***P≤0.001, ****P≤0.0001 (paired, two-sided Student's *t*-test). Data are mean±s.d. Scale bars: 50 µm.

We found that all *Sting^gt/gt^* dams exhibited an extensive decrease in size of their #3 mammary glands compared with WT counterparts following cross-fostering (Fig. S3B,C). Histological analysis of these glands via H&E staining revealed a vastly reduced quantity of lobuloalveoli (Fig. 6A), supported by a significant decrease in cellular content (Fig. 6B). A substantial increase in the cell death marker, c-CASP3, accounts for diminished MEC quantity within *Sting^gt/gt^* dams (Fig. 6C). Moreover, increased expression of pSTAT3 is indicative of premature involution within these glands, which also contributes to the decrease in overall cellular content, as the early phase of involution is characterized by massive cell death (Fig. 6D). Significantly decreased expression of pSTAT5 also contributes to the reduced quantity of milk-producing structures (Fig. 6E), although, this may be accounted for by the decrease in MECs available to produce lobuloalveoli. Taken together, these data substantiate the decrease in CSN2 expression within the #3 mammary glands of *Sting^gt/gt^* dams (Fig. 6F), ultimately, suggesting a failure of lactogenic differentiation. Of interest, we found that both E-cadherin (Fig. 6G) and K14 (Fig. 6H) were significantly expressed in the #3 glands of *Sting^gt/gt^* dams. Specifically, E-cadherin was expressed in a highly disorganized fashion, suggesting a basal-luminal phenotype that is associated with a pre-cancerous state (Gray et al., 2022). To determine whether the observed phenotype was related to impaired alveolar development within the early stages of postnatal mammary gland development, we evaluated #3 mammary glands between WT and *Sting^gt/gt^* mice in 10-week virgins. We found moderate differences in alveolar quantity within virgin glands; however, we did not observe notable differences in alveolar structure (Fig. 6I). Similarly, we noted no notable differences in alveolar structure in *Sting^gt/gt^* mice at pregnancy day 16, although, we found that the alveoli appeared to be modestly reduced in size (Fig. 6J). These findings parallel those observed previously in comparison of WT and *Sting^gt/gt^* #4 mammary glands; thus, we anticipate that additional contributors are likely to be involved in the lactational phenotype present in *Sting^gt/gt^* #3 mammary glands. Ultimately, our data suggest that STING is required for successful lactogenic differentiation, particularly within #3 mammary glands.

## DISCUSSION

Differentiated tissues rely upon both intrinsic signaling pathways and extrinsic environmental cues to facilitate development of the functional necessities of the specialized cell type. In response to hormone-induced transcriptional regulation, MECs undergo substantial metabolic reprogramming to support increased energetic and nutrient demand required for milk production. Communication between the mammary epithelium and immune cells within the stroma is also essential for successful differentiation of the gland (Coussens and Pollard, 2011; Hanasoge Somasundara et al., 2021; Watson et al., 2011). As MECs differentiate into milk-producing structures, they must adopt a partially suppressive microenvironment and become tolerogenic to rarely encountered milk proteins. A recent study has demonstrated that successful lactation is contingent upon diminished antigen-dependent T cell expansion (Betts et al., 2018). Thus, we anticipate that tumor cells can use intrinsic mechanisms of immune suppression during lactation to evade immune surveillance, ultimately promoting a pro-tumorigenic microenvironment. Mammary gland involution is known to exhibit an immunosuppressive phenotype to support tolerance to substantial cell death during tissue remodeling. The wound healing-like signature of involution has been widely associated with tumor promotion and progression (Borges et al., 2020; Fornetti et al., 2014; Hitchcock et al., 2022; Wallace et al., 2019); however, it has been proposed that lactation evolved as an inflammatory response to tissue damage and that specific inflammatory molecules have become vital mediators of lactation (Vorbach et al., 2006). This led us to hypothesize that lactation may be a novel driver of breast cancer initiation under conditions of failure to maintain cellular homeostasis. We predicted that investigating innate immune modulators within differentiating MECs would reveal previously unreported immune factors that respond to stress during lactation to support cellular differentiation and function. Our *in vitro* and *in vivo* models of lactogenic differentiation allowed us to identify STING as an immune mediator that responds to mitochondrial stress during lactation. From these studies, it is evident that STING activity supports maintenance of cellular homeostasis and the functional differentiation of MECs.

Canonically, STING is induced after cGAS activation following detection of cytosolic dsDNA, leading to production of type I IFNs and proinflammatory cytokines in an IRF3- and NF-κB-dependent manner, respectively. Here, we revealed that STING is induced during lactogenic differentiation in a cGAS-independent manner. We observed significant increases in gene expression of the type I IFNs, *Ifna4* and *Ifnb1* in response to differentiation cues. IFNα4 is involved in lymphocyte activation as well as anti-tumor immunity, whereas IFNβ balances expression of pro- and anti-inflammatory agents in addition to inhibiting T cell activation and proliferation (Boukhaled et al., 2021). This evidence supports a balanced inflammatory disposition during MEC differentiation to maintain cellular homeostasis and that the effector functions of immune cells are partially mediated by MECs and the cytokines they produce (Tower et al., 2022). Additionally, we illustrated a significant increase in gene expression of *Il1b* as well as *Tnf*. IL1β is a potent pro-inflammatory cytokine that is released, alongside IL18, upon activation of the NLRP3 inflammasome. Activation of the inflammasome occurs in response to NF-κB-mediated transcriptional regulation (Swanson et al., 2019; West, 2017). In addition, it has been established that STING facilitates NLRP3 localization to the ER, ultimately promoting inflammasome formation (Wang et al., 2020). Accumulation of mtROS has also been implicated in activation of the NLRP3 inflammasome (Chen et al., 2021; Jin et al., 2017; Martinon et al., 2009; Mittal et al., 2014; Swanson et al., 2019), suggesting that there may be multiple mechanisms of inflammasome activation throughout MEC differentiation resulting in a sterile inflammatory microenvironment. Moreover, both IL1β and TNF also exhibit wound healing attributes (Macleod et al., 2021), further suggesting that lactation serves as an insulting agent that has the potential to promote disease development when there is a failure to maintain homeostasis. In future investigations we will explore activation of inflammasome components to assess their contribution to lactogenic differentiation.

Maintenance of mitochondrial homeostasis is fundamental to mediate innate immune activation by limiting secretion of proinflammatory cytokines. One of the major mechanisms to sustain mitochondrial homeostasis is to eliminate damaged or fatigued mitochondria that release harmful ROS. We have previously illustrated that SIM2 directly interacts with and facilitates super complex formation to promote OXPHOS to enhance mitochondrial efficiency (Wall et al., 2023); thus, resulting in increased ROS production. Here, we demonstrated that a loss of *Sim2* confers a loss of STING activity. This occurs likely as a result of decreased mtROS accumulation, although additional mechanisms also appear to be at play. Specifically, we have evidence suggesting that SIM2 is involved in STING stabilization in a model of TNBC, as we found that STING protein expression is largely unaffected by CHX treatment when *SIM2* is overexpressed. Taken together, our findings lead us to anticipate that, under conditions of normal development, STING activity is required to maintain MEC function; however, in cancer, aberrant STING activity promotes tumorigenesis. Several lines of evidence support that STING becomes tumorigenic, although the switch that promotes this change requires further elucidation (Barber, 2015; Zhang et al., 2022). Our future studies will further uncover the mechanism(s) by which SIM2 regulates STING under normal developmental conditions as well as in cancer and assess the use of this relationship as a therapeutic benefit in breast cancer treatment options.

During postnatal mammary gland development, MECs upregulate several stress responses to support cell survival (Davis et al., 2016; Hadsell et al., 2011; Invernizzi et al., 2012; Sanchez et al., 2023; Shao and Zhao, 2014). Here, we show that MECs also upregulate STING during lactogenic differentiation and a loss of STING activity results in substantial deterioration of #3 mammary glands that resulted in decreased weight gain and fatality of foster pups. Although we noted modest alterations in the architectural development of the traditionally assessed inguinal #4 mammary glands of *Sting^gt/gt^* dams, these changes did not account for the significant decrease in pup weight or pup fatality observed. Previous reports led us to investigate the #3 mammary glands, in which we found a significant decrease in lobuloalveoli that are responsible for producing milk during lactation. Despite a modest decrease in alveolar quantity at an early developmental timepoint within the #3 mammary glands of *Sting^gt/gt^* mice, similar results were also noted in *Sting^gt/gt^* #4 mammary glands, ultimately suggesting additional contributors regarding the observed lactational phenotype within #3 mammary glands. Increases in pSTAT3 and c-CASP3, alongside a decrease in pSTAT5, were determined to account for reduced MEC quantity available to form lobuloalveoli. Under conditions of normal development, pSTAT5 expression, responsible for production of milk proteins and lobuloalveolar development, peaks during late pregnancy and throughout lactation and finally decreases during involution (Liu et al., 1995). *Stat5a* null mice were previously demonstrated to have fewer lobuloalveoli and demonstrated failure to lactate after the first gestational period (Liu et al., 1997). Accumulating evidence has revealed that downregulation of STAT5 during the reproductive cycle predisposes the mammary gland to tumorigenesis (Barash, 2006). This evidence suggests that decreased pSTAT5 in *Sting^gt/gt^* dams may influence cancer development. Furthermore, anomalous expression of pSTAT3 has been exposed as an early diagnostic marker for breast cancer and has also been demonstrated to promote metastasis and chemoresistance (Ma et al., 2020).

Traditionally, breast cancer has been categorized into four subtypes: luminal A, luminal B, HER2-enriched and basal-like (Yersal and Barutca, 2014). Luminal type tumors are responsive to endocrine treatment, whereas basal-like or TNBC tumors are the most resistant to chemotherapeutic intervention. Recently, evidence of breast cancers that possess both basal and luminal markers has been uncovered (Gray et al., 2022). Here, we found that the luminal epithelial cell marker, E-cadherin, is expressed both luminally and basally in the #3 mammary glands of *Sting^gt/gt^* dams. This acquirement of a basal-luminal phenotype signifies a pre-cancerous state, suggesting that STING is required to maintain homeostasis during lactogenic differentiation. Taken together, the expression profile and deterioration of #3 mammary glands accounts for fatality in pups nursed by *Sting^gt/gt^* dams, although the overall decrease in pup weight is likely attributable to variations in all mammary glands due to a loss of STING activity. Our findings support previous accounts that the postnatal development and maintenance of the various mammary gland pairs is unique and should be considered in further explorations into normal development, breast cancer initiation and metastasis (Veltmaat et al., 2013). In future investigations, we will employ use of a conditional knockout of *Sting* within the mammary gland to ameliorate any whole body affects or affects from a loss of *Sting* within surrounding immune cells. The stromal compartment of the mammary gland is established to contribute to successful development of the gland (Reed and Schwertfeger, 2010); thus, we will also assess differences in the immune milieu in #3 and #4 mammary glands.

Currently, morphological and gene expression differences between #3 and #4 mammary glands remain largely unexplored within the literature. We anticipate that studies into epithelial and stromal differences between #3 and #4 mammary glands in WT animals will further elucidate the mechanism that clarifies why, despite comparable differences between WT versus *Sting^gt/gt^* #4 mammary glands, only #3 mammary glands from *Sting^gt/gt^* mice undergo premature involution. Previous reports have demonstrated that homeobox (Hox) genes are involved in facilitating the maintenance of cell fate and identity in MECs (Chen and Sukumar, 2003). Furthermore, variations in Hox gene expression have been shown to differentially influence normal development between thoracic and inguinal mammary glands (Garcia-Gasca and Spyropoulos, 2000). These reports, in conjunction with more recent data, have illustrated a relationship between Hox gene signaling and STING activity (Li et al., 2023), highlighting an area of interest for future investigations regarding the observed phenotypic differences between #3 and #4 mammary glands in *Sting^gt/gt^* mice.

In conclusion, we have demonstrated that MECs upregulate STING to facilitate lactogenic differentiation of the developing mammary gland. We suggest that STING is activated in response to accumulating mtROS and that this occurs in a SIM2-dependent manner. Our findings establish that STING expression supports normal development of the mammary gland. We anticipate that these findings will elicit further investigations into STING activation during lactation to provide more comprehensive knowledge of its role(s) in normal development as well as breast cancer initiation and progression.

## MATERIALS AND METHODS

### Animals

All mice were cared for in accordance with the Texas A&M University ethical guidelines and Animal Care and Use Committee. All mice were housed under a standard 12 h light/12 h dark photoperiod and were provided access to food and water *ad libitum*. As this study looks at mammary gland development, only female mice were analyzed. Three mice were analyzed for each developmental time point. Mice were killed by CO_2_ asphyxiation followed by immediate cervical dislocation. The third anterior and fourth inguinal mammary glands were harvested where applicable. Glands were used for histological analysis as described below. *cGas*^−/−^ (JAX #026554; The Jackson Laboratory) and *Sting^gt/g^*^t^ (JAX #017537; The Jackson Laboratory) mice were generously provided by Dr A. Phillip West (The Jackson Laboratory, CT, USA). Cross-fostering studies were performed by replacing the litter of C57/B6J WT and *Sting^gt/gt^* mice with ten weight and aged-matched ICR donor pups to remove any potential bias from the original genotype of the pups. Average litter weights were recorded every day for 10 days. Dams were killed for tissue collection on lactation day 10 and #3 and #4 mammary glands were harvested for immunohistochemical analysis. Three mice were used for both WT and *Sting^gt/gt^* cross-foster collections.

### Immunohistochemical analysis

Mammary glands were harvested and immediately transferred to chilled 4% paraformaldehyde overnight. The following day, tissue was rinsed with 1× PBS and placed in 70% ethanol before being transferred to the Texas A&M University School of Veterinary Medicine and Biomedical Sciences histology core for paraffin embedding, sectioning and H&E staining. For immunohistochemical analysis, tissue sections were baked for 30 min at 60°C to dissolve paraffin, followed by further paraffinization in graded alcohols as follows: two washes in xylenes for 5 min each, two washes in 100% ethanol for 3 min each, one wash in each 95% ethanol and 70% ethanol, and finally 1× PBS. Antigen retrieval was achieved using a pressure cooker on high for 5 min with sections submerged in 10 mM sodium citrate (pH 6.0). Sections were then washed in 1× PBS for 5 min. To inhibit endogenous peroxidase activity, sections were incubated in 3% hydrogen peroxide for 6 min, followed by a 1× PBS wash. A PAP pen was then used to carefully circle the tissue before adding a blocking solution of PBS-T (1× PBS+Tween 20) containing 10% donor horse serum for 1 h. Following blocking, sections were incubated in primary antibody overnight at 4°C. The next day, sections were washed in PBS-T for 10 min before incubation in the appropriate secondary antibody (1:250) diluted in PBS-T containing 1% donor horse serum for 45 min. After incubation in secondary antibody, sections were washed in PBS-T for 5 min and then incubated for 30 min with avidin peroxidase using the Vectastain ABC Elite kit (Vector Laboratories, PK-6200). Full antibody details are in Table S3.

The chromogenic reaction was initiated using the DAB kit (Vector Laboratories, SK-4100) and then rinsed in dH_2_O for 5 min. Slides were then counterstained with methyl green [0.5 g methyl green in 100 ml 0.1 M sodium acetate buffer (1.36 g sodium acetate trihydrate, 100 ml H_2_O, pH 4.2, with glacial acetic acid)]. Following counterstain, slides were incubated in 95% ethanol for 3 min followed by ten dips in 100% ethanol and a 5-min incubation in xylenes. Slides were mounted using Permount Mounting Media (Sigma-Aldrich) and set to dry for at least 24 h before microscopic evaluation. Images were taken using a Zeiss AxioImager.v1 40× oil objective lens (25.2× magnification) in conjunction with Zeiss Axiovision software (Carl Zeiss Microscopy). QuPath v.0.4.2 was used to analyze images.

### Cell culture

CIT3 cells were generously provided by Dr Margaret Neville (University of Colorado Anschutz Medical Campus, CO, USA) and maintained in DMEM-F12 (Life Technologies, 11320082) supplemented with 2% fetal bovine serum (FBS; R&D Systems, S11550), 1% penicillin/streptomycin (Sigma-Aldrich, P4333-100ML), 10 µg/ml bovine insulin and 5 µg/ml mouse epidermal growth factor. Lactogenic differentiation was achieved by growing cells to confluency and incubating in differentiation media containing 2% FBS, 10 µg/ml bovine insulin, 3 µg/ml hydrocortisone and 3 µg/ml ovine prolactin. SUM159 cells were purchased from Asterand and grown in DMEM supplemented with 10% FBS and 100 units/ml penicillin/streptomycin. All cells were maintained in 5% CO_2_ at 37°C. CIT3 cells were dosed with 1 µM Mitoquinol (Cayman Chemical Company, 845959-55-9) or 100 µM Cycloheximide (Sigma-Aldrich, C7698-1G), with the addition of differentiation media (DMEM-F12 supplemented with 2% FBS, 10 µg/ml bovine insulin, 3 µg/ml hydrocortisone and 3 µg/ml ovine prolactin) and were harvested at relevant time points. SUM159 cells were dosed in DMEM supplemented with 10% FBS and harvested at relevant time points.

To generate the CIT3 *Sim2*^−/−^ cell line, the CRISPR/Cas9 *SIM2* KO vector {pLV[2CRISPR]-mCas9:T2A:Hygro-U6>(mSim2[gRNA#1])-U6>(mSim2[gRNA#2])} was designed with two gRNA sequences targeting exon 1 of m*Sim2* (gRNA#1 5′-AAAATGCGGCCAAAACCAGG-3′; gRNA#2 5′-CAGCTACCTGAAGATGCGCG-3′) and was generated and transformed in to Stbl3 competent cells by VectorBuilder. Lentiviral transduction of the CRISPR/Cas9 *SIM2* KO vector were performed as previously described (Kwak et al., 2007).

For transient transfection of dsDNA fragments (dsDNA sequence can be found in Table S1), dsDNA oligos were prepared as follows: 5 µl of 3 µg/µl dsDNA-F and dsDNA-R were added to 5 µl annealing buffer (10 mM Tris-HCl pH 7.5, 1 mM EDTA and 50 mM NaCl) and nuclease free water for a total volume of 50 µl. Reaction tubes were incubated in a thermal cycler (Bio-Rad, T100 Thermal Cycler) under the following conditions: 30 min at 37°C followed by 5 min at 95°C. Samples were stored at 4°C. A ratio of 2:1 Gene Juice Transfection Reagent (EMD Millipore, 70967-3): µg nucleic acid was used. A total of 2 µg of DNA was transfected into cells seeded at ∼70% confluency on 60×15 mm cell culture plates. Appropriate amounts of Opti-MEM and Gene Juice Transfection Reagent were incubated for 5 min at room temperature. Next, 2 µg of DNA was added, and the mixture was incubated for 15 min at room temperature. Following the incubation period, the DNA mixture was added dropwise to cells. Samples were incubated for 2-8 h before harvesting.

For staining of cellular ROS, cells were plated in a 96-well plate and incubated in 2 µl/ml CellRox reagent (Thermo Fisher Scientific, C10422) for 30 mins. Cells were then washed with PBS, and fresh PBS was added to measure fluorescence intensity on a plate reader. PBS was removed and cells were incubated with 0.8 µl/ml Hoechst 33342 (Thermo Fisher Scientific, 62249) for 10 min. Cells were washed with PBS and fluorescence intensity was measured on a plate reader. The relative fluorescence intensity of cellular ROS was normalized to Hoechst 33342.

### RNA isolation and qPCR

Cells were washed with ice-cold PBS and RNA was isolated using the High Pure RNA Isolation Kit (Roche Applied Science) adhering to the manufacturer's protocol for centrifugal isolation, inclusive of DNAse digestion of RNA contaminants. Final RNA was eluted in 50 µl of High Pure RNA Isolation Elution Buffer. Depending on the concentration of the RNA sample, 0.2-1 µg of RNA was used for reverse transcription reactions. cDNA was produced using the iScript cDNA Synthesis kit (Bio-Rad) adhering to the manufacturer's protocol: each sample received 4 µl of 5× iScript Reaction Mix and 1 µl of iScript Reverse Transcriptase. Reaction tubes were briefly spun down and incubated in a thermal cycler (Bio-Rad, T100 Thermal Cycler) under the following conditions: 5 min priming at 25°C, 20 min of reverse transcription at 46°C, and finally 1 min of reverse transcription inactivation at 95°C. Samples were held at 4°C. In a 384-well plate (Bio-Rad, HSR4801), 4 µl of each cDNA sample was mixed with 5 µl of iTaq Universal SYBR Green Supermix (Applied Biosystems, C14512) and 1 µl of primer. A total of three biological replicates and two technical replicates were run per sample. Reactions were carried out using the following cycle conditions: 95°C for 2 min, 95°C for 15 s and 60°C for 1 min (plate read, repeat 39 times), 95°C for 10 s, 65°C for 31 s, then 65°C for 5 s (+0.5°C/cycle, ramp 0.5°C/s) plate read (repeat, 60 times). Relative expression analysis was determined using the ddCT method relative to the control sample. Primers used for qPCR analysis can be found in Table S2.

### Immunoblotting

Cells were washed with 3 ml of chilled 1× PBS to remove dead cells and excess media. Then, 1 ml of 1× PBS was added directly to cells for scraping. The PBS-cell mixture was transferred to a 1.5 ml BioRuptor tube (C30010016) and spun at 1500 rpm (245 ***g***) for 10 min at 4°C. RIPA buffer (Thermo Fisher Scientific, 89900) containing protease and phosphatase inhibitors was used to lyse cells. Samples were vortexed for 15 s to facilitate lysing and were chilled on ice for 10 min. To further assist in cell lysis, all samples were sonicated using a BioRuptor Pico (B01060010) adhering to the following protocol: vortex samples, 30 s on, 30 s off for two cycles, remove samples to briefly vortex, followed by 30 s on, 30 s off for one additional cycle. After sonication, supernatant was transferred to a sterile 1.5 ml microcentrifuge tube and spun at max speed (12,700 rpm; 18,000 ***g***) for 15 min. Supernatant was then transferred to another 1.5 ml microcentrifuge tube and protein samples were stored at −80°C. Protein concentrations were determined using a DC Protein Assay (Bio-Rad, 500-0116). For SDS-PAGE analysis, 40 μg of protein sample was loaded into acrylamide gels ranging from 8-15%. Contingent upon total volume need for loading, an appropriate volume of 6× loading buffer (60% glycerol, 0.3 M Tris-HCl pH 6.8, 12 mM EDTA, 12% SDS, 6% β-mercaptoethanol, 0.5% bromophenol blue) was added to samples before boiling at 100°C for 5 min. Before loading the gels, samples were quick spun using a table-top centrifuge. Gels were run using 1× TGS Running Buffer (Bio-Rad) at 90 V until samples made it through the stacking gel and then at 120 V until samples reached the end of the gel. Gels were transferred onto PDVF membranes in 1× transfer buffer [800 ml Milli-Q H_2_O, 100 ml 10× Transfer buffer (144 g glycine+30.28 g Tris base) and 100 ml methanol] at 90 V for 90 min. Membranes were then blocked in 5% milk (Great Value, Instant Non-Fat Dry Milk) in TBS-T (1× TBS+0.05% Tween 20) at room temperature, rocking for 60 min. After blocking, membranes were rinsed in TBS-T before primary antibody incubation at 4°C overnight while rocking. The next day, membranes were washed while rocking in TBS-T three times for 10 min each before incubation with the appropriate secondary antibody for 60 min at room temperature. Blots were washed again as previously described before visualization using a ChemiDOC MP Imaging System (Bio-Rad) following a 5 min incubation with ProSignal Pico ECL Spray (Genesee Scientific, 20-300S). A list of antibody sources and dilutions used can be found in Table S3.

### Isolation of mitochondria and mitochondria-associated membranes

The protocol from Wieckowski et al. was used to isolate mitochondria and mitochondria-associated membranes (MAMs) from cells (Wieckowski et al., 2009). To begin, media was removed and cells were washed with 7 ml of ice-cold 1× PBS. After washing, 3 ml of trypsin were added to each plate and cells were incubated at 37°C for 5-10 min to allow cells to detach. Cellular suspension was then transferred to a 50 ml conical tube and centrifuged at 600 ***g*** for 5 min at 4°C. The supernatant was discarded, and cells were resuspended in 30 ml 1× PBS. Cells were spun again at 600 ***g*** for 5 min at 4°C. PBS was discarded, and the cell pellet was resuspended in 15 ml of cold IB buffer [150 ml of starting buffer (20.5 g of mannitol, 13 g of sucrose in 400 ml of MilliQ H_2_O, 15 ml of Tris HCl; pH to 7.4 and bring up to 500 ml) and 60 µl of 100 mM EGTA pH 7.4 (3.8 g of EGTA in 100 ml MilliQ H_2_O)]. Cells were homogenized using a 15 ml dounce 35× and transferred to a 50 ml conical tube. Cellular suspension was centrifuged at 600 ***g*** for 5 min at 4°C. Supernatant was collected in a separate 50 ml conical tube labeled ‘crude mitochondria’. Cellular pellet was resuspended in 7 ml of IB buffer and dounced and centrifuged as previously described. This was repeated approximately five times. After final centrifugation, the pellet (unbroken cells and nuclei) was resuspended in 1 ml of GRO buffer (2 mM MgCl_2_, 3 mM CaCl_2_, 10 mM Tris-HCl, 5 mM HEPES, 0.5% NP-40) and stored at −80°C. The crude mitochondrial supernatant was then centrifuged at 7000 ***g*** for 10 min at 4°C. The supernatant was collected in a separate tube labeled ‘microsome’. The crude mitochondrial pellet was resuspended in 10 ml of cold IB buffer and centrifuged at 7000 ***g*** for 10 min at 4°C. The supernatant was discarded and the mitochondrial pellet was again resuspended in 10 ml of cold IB buffer and centrifuged at 7000 ***g*** for 10 min at 4°C (pre-cool the ultra-centrifuge during this step). Again, the supernatant was discarded, and the crude mitochondrial pellet was resuspended in 3 ml of cold MRB buffer (9.12 g of mannitol, 2 ml of 0.5 M HEPES pH 7.4, 1 ml of 100 mM EGTA, pH to 7.4, final volume 200 ml) using wide bore pipette tips. Approximately 100 µl of this crude mitochondrial suspension was collected in a separate 1.5 ml tube and stored at −80°C. Following crude mitochondrial resuspension, 16 ml of Percoll medium (basal solution: 2.052 g of mannitol, 500 µl of 100 mM EGTA, pH 7.4, final volume 35 ml; 4.8 ml Percoll (Sigma-Aldrich, P1644-100ML)+11.2 ml basal solution) was added to a 14 ml thin-wall polyallomer ultra-centrifuge tube. Crude mitochondrial suspension was layered dropwise on top of the Percoll medium. MRB buffer was then layered (dropwise) on top of the crude mitochondrial suspension, leaving ∼4-5 mm of space from the top of the tube. Where there was only one sample, the appropriate balance was weighed with water. Tubes were centrifuged at 34,000 rpm (95,000 ***g***) for 30 min at 4°C. While the samples were spinning, Milli-pore protein concentrators were used to concentrate microsome sample. Then 500 µl of sample was added to the tube and it was spun at 14,000 ***g*** for 15 min at room temperature. The concentrated sample was collected in a separate 1.5 ml tube labeled microsome. This process was repeated until ∼100 µl was collected. The microsome sample was stored at −80°C. Following ultra-centrifugation, the MAM (top) and mitochondrial (bottom) fractions had separated, and a 1 ml micropipette was used to collect fractions, which were diluted approximately five times with MRB buffer in separate 50 ml conical tubes. Mitochondrial and MAM fractions were centrifuged at 7000 ***g*** for 10 min at 4°C. The MAM supernatant was transferred to an ultra-centrifuge tube and centrifuged at 35,000 rpm (100,000 ***g***) for 1 h at 4°C. Some of the MRB/Percoll mixture was aspirated from the mitochondrial pellet and more MRB buffer added. Centrifugation was repeated at 7000 ***g*** for 10 min at 4°C. we repeated aspirating Percoll and adding more MRB about five times until supernatant appeared clear. Once supernatant was clear and there was a decent mitochondrial pellet, MRB buffer was aspirated, leaving ∼100 µl to resuspend pellet in. The pure mitochondrial pellet was transferred into a separate 1.5 ml tube and stored at −80°C. Once the MAM suspension was finished, the MAMs were picked up with 200 µl micropipettes and transferred to 1.5 ml tubes and stored at −80°C.

### Nuclear isolation from crude pellet

The nuclear pellet was diluted with ∼3 ml of GRO buffer and transferred to a 7 ml dounce. Tight junction was used to dounce samples ∼30 times. The solution was poured through a cell strainer into a 50 ml conical tube, the strainer removed and cap replaced. Samples were centrifuged at 500 ***g*** for 5 min at 4°C. Supernatant was discarded, and the nuclear pellet was resuspended in IB buffer. Samples were stored at −80°C.

### Statistical analysis

All experiments were carried out in a minimum of biological triplicates and technical duplicates and each comparison was performed in a minimum of two independent assays. Bar graphs were plotted using the mean±s.d. Statistical analyses were performed using Prism 9 software. Significant differences between continuous variables were assessed using paired, two-sided Student's *t*-tests and a *P*-value ≤0.05 was considered significant unless otherwise noted.

## Supplementary Material

10.1242/develop.202998_sup1 Supplementary information
