# Supplementary material for: Loss of STING impairs lactogenic differentiation
Source: Development. 2024 Oct 14;151(19):dev202998. doi: 10.1242/dev.202998 (PMC11528151; doi:10.1242/dev.202998)
Supplement: Supplementary information [file develop-151-202998-s1.pdf]

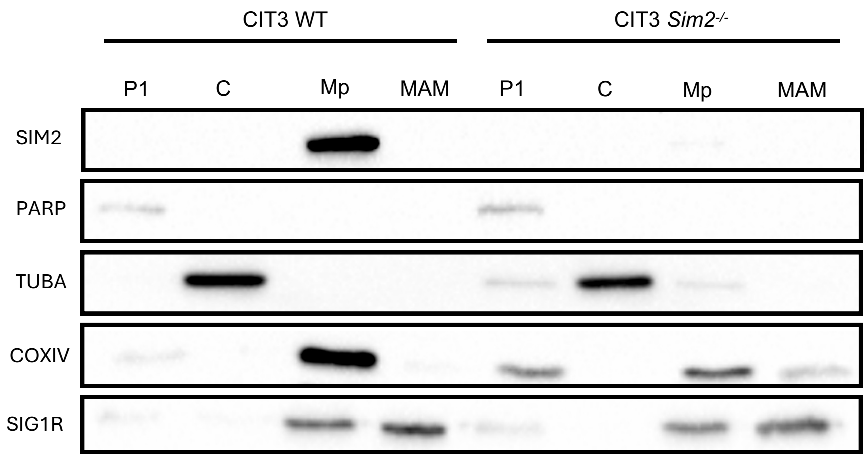

Fig. S1. Validation of SIM2 loss. Immunoblot analysis of SIM2 following subcellular fractionation. P1 – nucleus and unbroken cells, C - Microsome (plasma membrane, lysosome, ER, cytosol), Mp – pure mitochondria, MAM – mitochondria associated membrane.

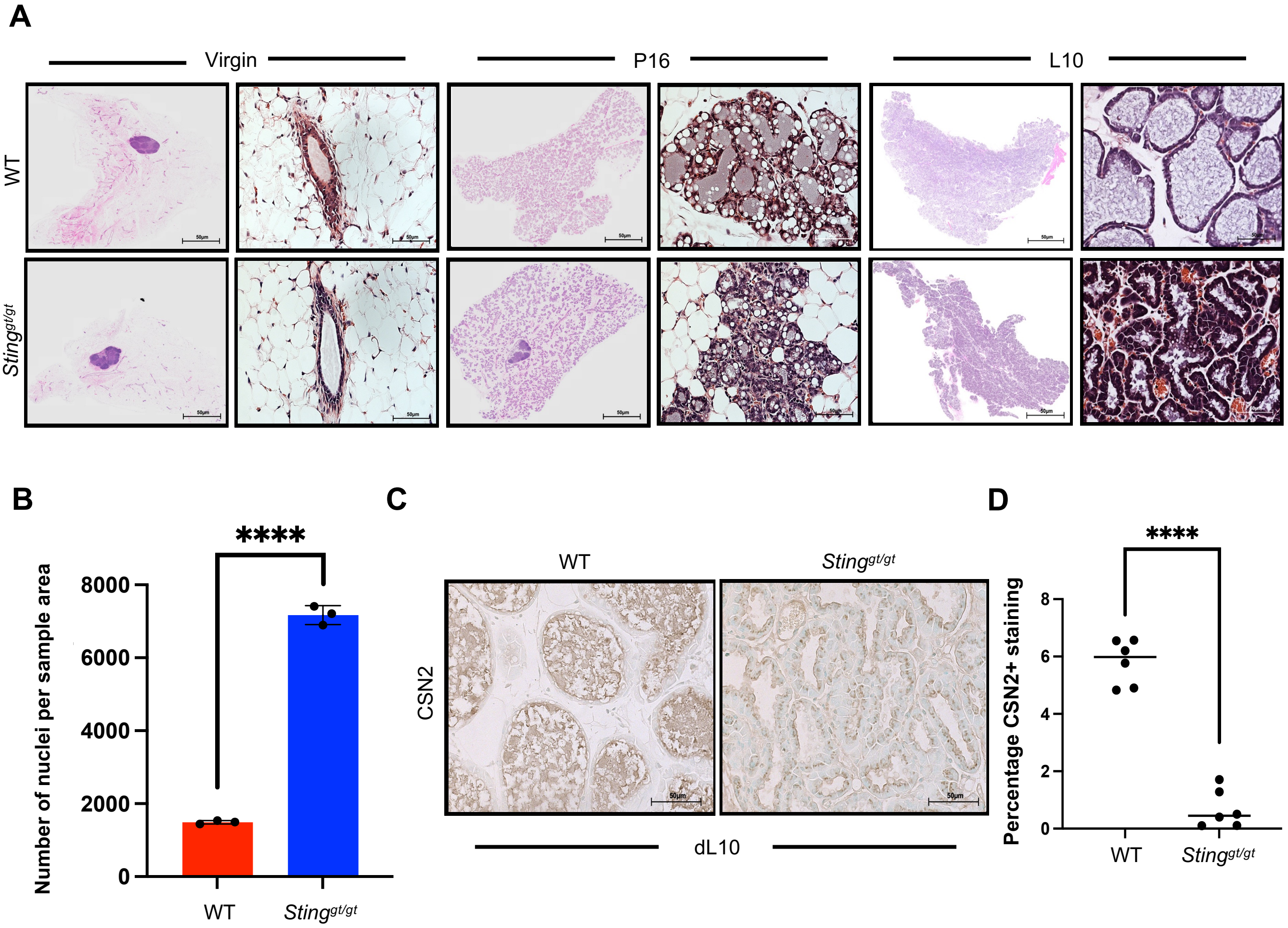

Fig. S2. The *Sting*$^{gt/gt}$ during lactation. A H&E-stained #4 mammary tissue sections from WT and *Sting*$^{gt/gt}$ mice at 10-week virgin, pregnancy day 16 and lactation day 10. **B** Cellularity quantification from lactation day 10. **C** Immunostaining for CSN2 in WT and *Sting*$^{gt/gt}$ mice at lactation day 10. **D** Quantification of CSN2 positive staining. ****p ≤ 0.0001.

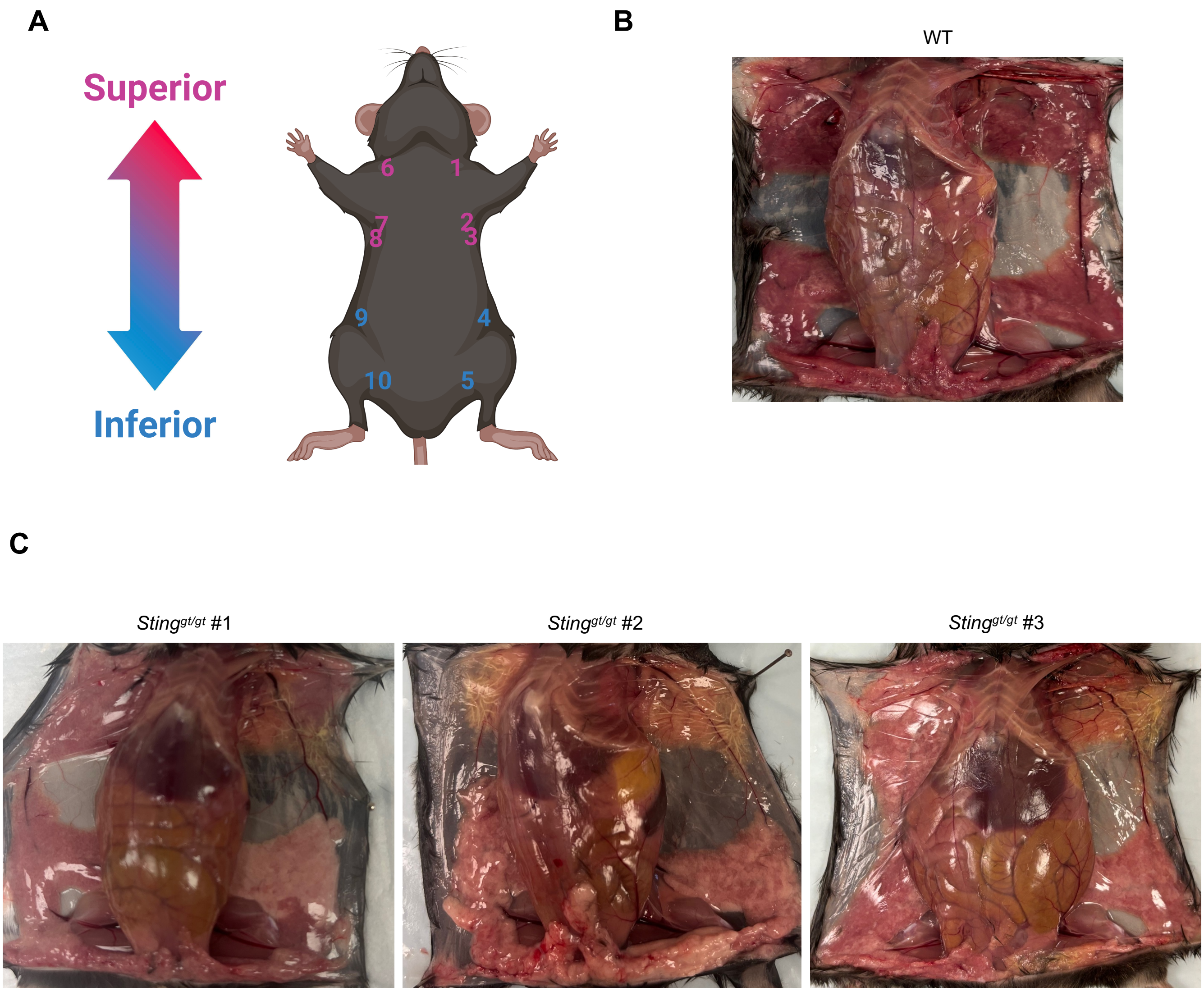

Fig. S3. Lactational disruption of #3 mammary glands in *Sting$^{gt/gt}$* dams. **A** Model of murine mammary gland positions. Pictures of mammary glands at lactation day 10 following cross-fostering in **B** WT and **C** *Sting$^{gt/gt}$* dams.

Table S1. dsDNA sequence

Forward	Reverse
TACAGATCTACTAGTGATCTATGACTGAT CTGTACATGATCTACATACATACAGATCT ACTAGTGATCTATGACTGATCTGTACATG ATCTACA	TGTAGATCATGTACAGATCAGTCATA GATCACTAGTAGATCTGTATGTATGT AGATCATGTACAGATCAGTCATAGAT CAC TAGTAGATCTGTA

Table S2. Primer list

Target	Forward	Reverse
mCsn2	TGTGCTCCAGGCTAAAG TTCACT	GGTTTGAGCCTGAGCATATGG
mIfnα4	CTTTCCTCATGATCCTGG TAATGAT	AATCCAAAATCCTTCCTGTCCTTC
mIfnβ	CCCTATGGAGATGACGG AGA	CCCAGTGCTGGAGAAATTGT
mIL-1β	TGCCACCTTTTGACAGT GATG	TGCCACCTTTTGACAGTGATG
mTnf	ATCCGCGACGTGGAACT G	ACCGCCTGGAGTTCTGGAA
mIL-6	TGATGCACTTGCAGAAA ACA	ACCAGAGGAAATTTTCAATAGGC
mSting	CCGATTTCCGGGGGATC AAT	GCAAGTGGCTTCTGAATGGG
mHprt	CTAGTCCTGTGGCCATCT GC	ATCAAAAGTCTGGGGACGCA
mActb	GCAACGAGCGGTTCCG	CCCAAGGAAGGCTGGA
hSTING	CATGGGCGTCTCTGGTC ATATTACATCGGATATC TGCGGCTG	TGGTACCGTCTCGGATCCAGAGAAAT CCGTGCGGAGAGGGAGGGGCT
hTBP	CGTCCCAGCAGGCAACA	GGTGCAGTTGTGAGAGTCTGTGA

Development • Supplementary information

Table S3. Antibody list

Antibody	Dilution	Manufacturer	Catalog No.
STING	WB 1:500<br>IHC 1:100	Cell Signaling Technology	13647S
cGAS	WB 1:500	Cell Signaling Technology	15102S
SIM2	WB 1:500	Abcam	ab131161
CSN2	IHC 1:100	Santa Cruz Biotechnology	sc-166530
pSTAT3	IHC 1:250	Cell Signaling Technology	9131
pSTAT5	IHC 1:250	Cell Signaling Technology	9359S
E-cadherin	IHC 1:150	R&D Systems	AF748
Cytokeratin 14 (K14)	IHC 1:50	OriGene Technologies	BP5009
c-CASP3	IHC 1:100	Cell Signaling Technology	9661S
Actb	WB 1:1000	Cell Signaling Technology	37005
Anti-mouse IgG, HRP-linked	WB 1:5000	Cell Signaling Technology	7076S
Anti-rabbit IgG, HRP-linked	WB 1:5000	Cell Signaling Technology	7074S
Anti-mouse biotinylated	IHC 1:250	Vector Laboratories	BMK-2202
Anti-rabbit biotinylated	IHC 1:250	Vector Laboratories	BA-1000
Anti-guinea pig biotinylated	IHC 1:250	Vector Laboratories	BA-7000
